# Intraoperative Techniques Aiding Margin Assessment and Nerve Sparing During Robot-Assisted Radical Prostatectomy

**DOI:** 10.3390/cancers18162645

**Published:** 2026-08-17

**Authors:** Mikołaj Kisiała, Zuzanna Czyczerska, Jakub Kowalski, Wojciech Grzelczak, Mateusz Sobala, Jakub Karwacki, Michał Tulski, Dominik Zawadzki, Patryk Patrzałek, Izabela Skowron, Jan Łaszkiewicz, Wojciech Tomczak, Wojciech Krajewski, Tomasz Szydełko, Bartosz Małkiewicz

**Affiliations:** 1 Department of Minimally Invasive and Robotic Urology, University Center of Excellence in Urology, Wroclaw Medical University, 50-556 Wroclaw, Poland; 2 Department of Pathophysiology, Wroclaw Medical University, 50-368 Wroclaw, Poland

**Keywords:** prostate cancer, robot-assisted radical prostatectomy, positive surgical margins, intraoperative margin assessment, nerve-sparing surgery, NeuroSAFE

## Abstract

Robot-assisted radical prostatectomy (RARP) requires a balance between complete cancer excision and preservation of urinary continence and erectile function. Because the preservation of neurovascular bundles adjacent to the prostate surface contributes to postoperative functional recovery, intraoperative margin assessment and surgeon-guidance technologies may help refine resection and support individualized nerve-sparing decision-making. Neurovascular structure-adjacent frozen-section examination remains the most studied and clinically established method, with evidence supporting reduced positive surgical margins and broader nerve preservation, but its use is limited by cost, workflow complexity, and pathology infrastructure. Fluorescence confocal microscopy is promising but remains limited by short and apical margins; its improved accuracy for longer margins raises concerns about its reliability for routine intraoperative margin assessment. Other optical, spectroscopic, and prostate-specific membrane antigen-targeted molecular margin assessment techniques have demonstrated inconsistent diagnostic performance, with particularly low sensitivity for assessment approaches remain investigational, whereas intraoperative ultrasound, three-dimensional and augmented-reality models, fluorescence guidance, and image-enhancement systems should be viewed as surgical guidance tools rather than direct margin-assessment methods. Intraoperative technologies may improve surgical decision-making during RARP, but prospective multicenter studies with standardized oncological and functional endpoints are needed before broader adoption.

## Simple Summary

Robot-assisted surgery for prostate cancer aims to remove the tumor completely while preserving nearby nerves when this can be done safely. However, nerve preservation is not appropriate in every patient, especially when the cancer is suspected to extend beyond the prostate or close to the surgical edge. In these situations, wider tissue removal, including part or all of the nearby nerve structures, may be necessary to achieve adequate cancer control. This review examines techniques that can help surgeons assess the surgical margin during the operation and choose the most appropriate extent of tissue removal. It also discusses imaging and navigation tools that may support these decisions. By comparing established and emerging approaches, the review highlights which methods are most clinically useful and where further research is needed to improve both cancer control and postoperative functional outcomes.

## 1. Introduction

Robot-assisted radical prostatectomy (RARP) is the treatment of choice for patients with localized and selected locally advanced prostate cancer (PCa) [1]. If oncologically feasible, RARP seeks to achieve complete prostate excision while preserving urinary continence and erectile function. Because the neurovascular bundles (NVBs) lie close to the posterolateral aspect of the prostate, surgeons must balance nerve preservation against the risk of positive surgical margins (PSMs), which are associated with a higher risk of biochemical recurrence and the need for adjuvant or salvage treatment [2]. Currently, surgical planning relies on preoperative decision-making, which relies on clinical variables, biopsy findings, nomograms, and multiparametric magnetic resonance imaging (mpMRI). The European Association of Urology (EAU) guidelines recommend offering nerve-sparing (NS) surgery only on the side with a low risk of extracapsular disease [1]. However, preoperative prediction remains imperfect; clinical and radiological factors are generally poor predictors of extraprostatic extension (EPE), and external validation of mpMRI-inclusive, side-specific EPE nomograms has shown only moderate discrimination [3]. Moreover, PSM rates after RARP remain substantial and vary according to patient and disease characteristics, with large multicenter studies reporting overall rates of 15.7% and 29.9% [2,4]. Thus, intraoperative margin assessment and surgeon-guidance techniques that provide real-time information on margin status or NVB involvement become increasingly relevant.

Currently, the most established and frequently used intraoperative margin assessment and nerve-sparing (NS) technique is neurovascular structure-adjacent frozen-section examination (NeuroSAFE), a method based on frozen-section analysis of the excised prostate specimen from the posterolateral side adjacent to NVBs. Other approaches seek to provide faster, digital, and less destructive tissue assessment. Such methods include fluorescence confocal microscopy (FCM), prostate-specific membrane antigen (PSMA)-targeted molecular imaging, structured illumination microscopy (SIM), Raman histology, optical coherence tomography (OCT), reflectance spectroscopy, and multiphoton microscopy (MPM). In parallel, other technologies do not directly assess the surgical margin but instead guide the surgeon during dissection. These include intraoperative ultrasonography, three-dimensional (3D) intraoperative models, augmented reality (AR) navigation, fluorescence guidance, and image-enhancement techniques.

This narrative review summarizes current intraoperative techniques used to assess surgical margins or guide NS during RARP, with emphasis on feasibility, diagnostic performance, and oncological and functional outcomes. For the purposes of this review, techniques were classified according to their primary intraoperative function. Direct intraoperative margin-assessment techniques were defined as methods that interrogate excised prostate tissue or the surgical margin itself for the presence of tumor, irrespective of whether the entire specimen surface is assessed. In contrast, surgeon-guidance tools provide anatomic or tumor-localization information to influence the dissection plane but do not directly determine the histologic, optical, or molecular status of the surgical margin. Accordingly, a demonstrated effect on PSM rates was not used as the criterion for classification.

Recent comprehensive reviews have highlighted the rapid evolution of these real-time intraoperative modalities [5]. This review is designed to provide a practical RARP-oriented synthesis that separates direct intraoperative margin-assessment methods from surgeon-guidance tools and emphasizes the translational maturity of each approach. Particular attention is given to recent evidence published in 2025–2026 and to the question of which technologies are realistically closest to clinical implementation.

## 2. Methods

We prepared this pragmatic narrative review in accordance with the Scale for the Assessment of Narrative Review Articles (SANRA) checklist [6]. A structured literature search was performed in PubMed, Embase, and Web of Science. The final database search was executed on 31 March 2026 and included studies published from database inception up to that date.

The search strategy combined controlled vocabulary and free-text terms related to prostate cancer, radical prostatectomy, robot-assisted radical prostatectomy, nerve-sparing surgery, neurovascular bundles, frozen-section analysis, surgical margins, intraoperative margin assessment, intraoperative guidance, and the landmark artery. The following search strategy was used in PubMed: ((“Prostatic Neoplasms”[MeSH Terms] OR “prostate cancer”[Title/Abstract]) AND (“radical prostatectomy”[Title/Abstract] OR “robot-assisted radical prostatectomy”[Title/Abstract] OR “robotic radical prostatectomy”[Title/Abstract] OR RARP[Title/Abstract]) AND (“nerve sparing”[Title/Abstract] OR “nerve-sparing”[Title/Abstract] OR “neurovascular bundle*”[Title/Abstract] OR “frozen section*”[Title/Abstract] OR “intraoperative margin*”[Title/Abstract] OR “margin assessment”[Title/Abstract] OR “intraoperative guidance”[Title/Abstract] OR “landmark artery”[Title/Abstract: ~0])). The search syntax was adapted to the indexing and search conventions of Embase and Web of Science. After removal of duplicates, 354 records underwent title and abstract screening, of which 102 articles were assessed in full text.

Because this was a narrative review encompassing both clinically applied intraoperative techniques and the methodological foundations of emerging technologies, the structured database search was supplemented by backward citation searching of eligible studies and relevant review articles. Reference lists were examined iteratively to identify additional studies and foundational methodological publications that did not necessarily contain prostatectomy-specific terminology and would therefore be unlikely to be retrieved by the focused primary search. Where appropriate, additional publications were identified through citation tracking in Web of Science and by searching the original journal or publisher source.

Study selection was performed by J.Ko. and M.S. Two reviewers independently assessed potentially eligible studies, and disagreements were resolved by discussion and consensus. In total, 65 publications were retained for the narrative synthesis presented in Section 3 and Section 4. Original articles, comparative studies, feasibility studies, and relevant review articles in English were considered. We gave preference to studies reporting diagnostic performance, intraoperative feasibility, effects on nerve-sparing decisions, surgical margin outcomes, and postoperative functional or oncological outcomes. The aim was not to perform a formal systematic review, but to provide a clinically oriented synthesis of technologies relevant to intraoperative decision-making during RARP. No formal protocol registration or risk-of-bias assessment was performed.

## 3. Intraoperative Margin Assessment Tools

### 3.1. NeuroSAFE

**Figure 1:**
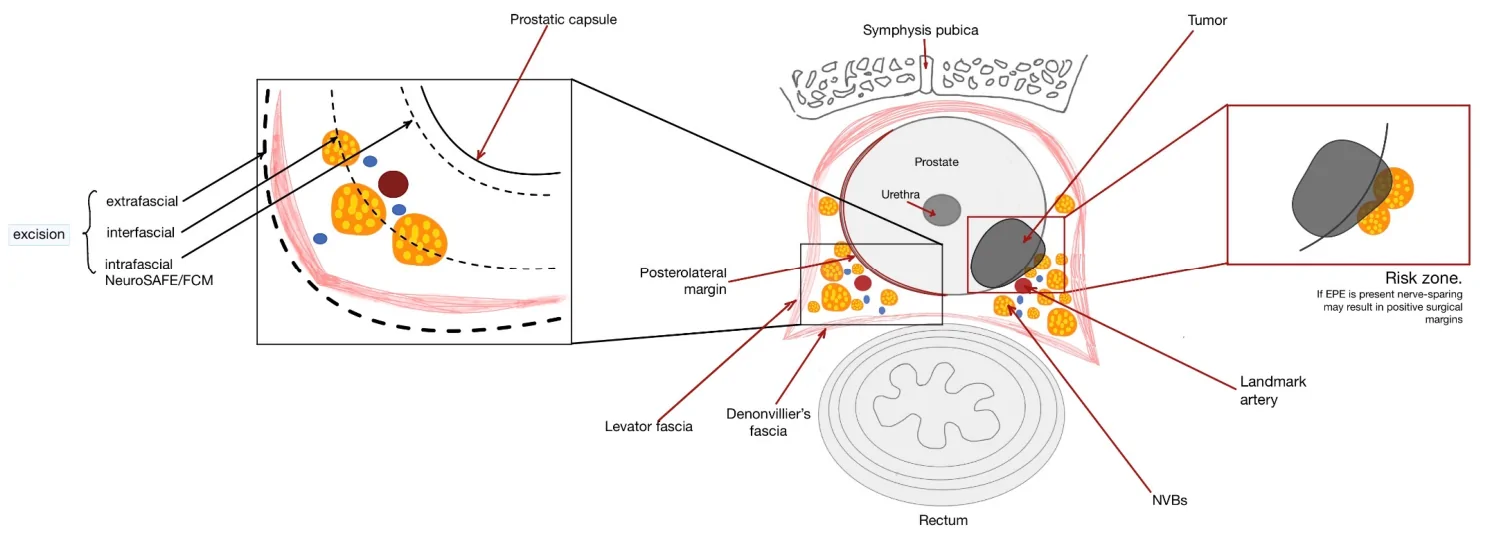
Schematic pelvic cross-section illustrating the anatomical basis for intraoperative margin assessment during nerve-sparing RARP. The figure shows the prostate and adjacent structures, including the neurovascular bundles, levator fascia, Denonvilliers’ fascia, and colon. During NeuroSAFE or FCM/LaserSAFE, an intrafascial dissection plane may be selected to preserve the neurovascular bundles. However, when the tumor extends beyond the prostatic capsule toward the posterolateral margin or neurovascular bundle, nerve-sparing dissection without intraoperative margin assessment may leave residual cancer cells behind, resulting in a positive surgical margin.

**Figure 2:**
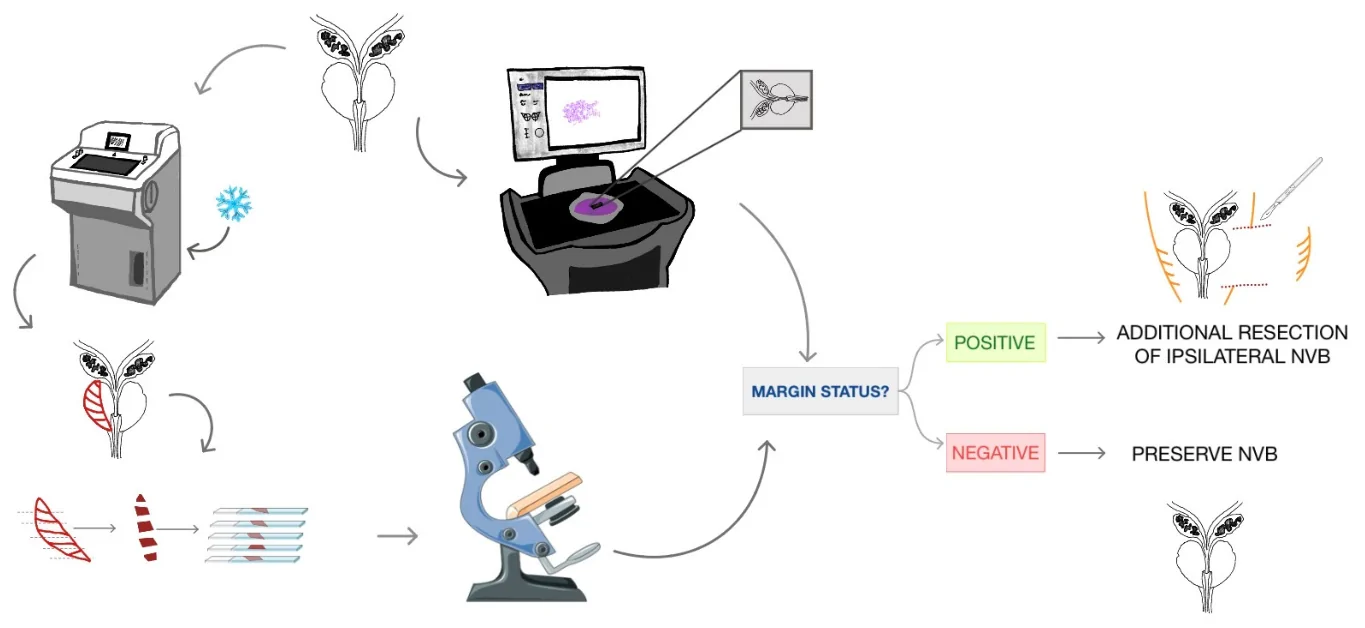
Schematic workflow of intraoperative margin assessment during nerve-sparing RARP using NeuroSAFE or FCM/LaserSAFE. After prostate excision, the margin adjacent to the neurovascular bundle is assessed to guide whether the nerves can be safely preserved. In the NeuroSAFE pathway, tissue from the posterolateral surface of the prostate is removed, frozen, sectioned, stained, and examined microscopically by a pathologist. In the FCM/LaserSAFE pathway, fresh tissue is stained and scanned using confocal microscopy, either as shaved margins or by en face assessment of the intact specimen, depending on the platform. If the margin is positive, additional ipsilateral neurovascular bundle resection is performed. If the margin is negative, the neurovascular bundle is preserved. Adapted from Servier Medical Art (https://smart.servier.com, accessed on 27 May 2026), licensed under CC BY 4.0 (https://creativecommons.org/licenses/by/4.0/, accessed on 27 May 2026).

NeuroSAFE is an intraoperative technique aimed at assisting NS during radical prostatectomy (RP). Its main objective is to maximize preservation of NVBs without compromising oncological safety. In an original description of NeuroSAFE by Martini Clinic [7], the surgeon performs bilateral intrafascial NS RP, and then the prostatic tissue adjacent to the NVB is dissected into wedge shapes from base to apex. The surgeon then inks the inner and outer surgical margins in different colors and marks the apical end to preserve orientation. The specimen is then sent for histopathological examination, sliced into 3–4 mm sections, producing roughly 10–25 tissue blocks per patient; these are embedded, frozen, stained with hematoxylin and eosin (H&E), and reviewed by a genitourinary pathologist (Figure 1). A margin is called positive if even one invasive malignant gland or cell touches the inked outer margin. While pathology is processing the sample, the surgeon continues with other steps such as hemostasis, bladder closure, placement of anastomotic sutures, and lymph-node dissection, so the average pathology turnaround of about 35 min does not interfere with surgical procedures [7]. If NeuroSAFE is positive, the surgeon performs an ipsilateral secondary neurovascular resection, including the rectolateral part of Denonvilliers’ fascia, before completing the anastomosis (Figure 2); that secondary specimen is then sent separately for final pathology. The overall margin status is based on the entire prostatectomy specimen, including the NeuroSAFE sections [7,8,9].

Among intraoperative margin-control strategies used during RARP, NeuroSAFE remains the most extensively studied and the most clinically established. Available evidence, derived mainly from retrospective cohort studies but supported by a randomized trial, suggests that NeuroSAFE can increase nerve-sparing rates and improve margin control, with no clear evidence of worse biochemical recurrence outcomes [10]. However, interpretation should be cautious, as much of the comparative literature relies on historical controls and is influenced by differences in baseline tumor stage and case mix [11,12,13,14,15]. The pivotal multicenter, patient-blinded, randomized phase 3 NeuroSAFE PROOF trial evaluated erectile function at 12 months as its primary endpoint using the IIEF-5. After a median follow-up of 12.3 months, mean IIEF-5 scores were significantly higher in the NeuroSAFE group than in the standard RARP group (12.7 vs. 9.7; adjusted mean difference 3.18, 95% CI 1.62–4.75; *p* < 0.0001). Urinary continence was significantly better at 3 months (adjusted ICIQ mean difference −1.41, 95% CI −2.42 to −0.41; *p* = 0.006), but this difference was no longer significant at 6 months (adjusted mean difference −0.37, 95% CI −1.35 to 0.62; *p* = 0.46) [11]. These results support an improvement in erectile function at 12 months, whereas the continence benefit appears to be limited to the early postoperative period. NeuroSAFE PROOF provides evidence for functional benefit, but its implications for long-term oncological outcomes remain uncertain.

Surgical margin status is a pathological surrogate and should not be considered equivalent to clinically relevant oncological outcomes such as biochemical recurrence or survival. Moreover, oncological follow-up in NeuroSAFE PROOF is currently limited to approximately 12 months, and longer prospective follow-up is needed to establish long-term oncological safety.

Because of the nature of the intervention, the operating teams could not be blinded to treatment allocation, which introduces the possibility of performance bias. In addition, the trial was conducted by experienced RARP surgeons, and the influence of the NeuroSAFE implementation learning curve on outcomes in less experienced centers remains uncertain.

Despite its clinical maturity, NeuroSAFE remains constrained by several factors, such as pathology infrastructure requirements, workflow complexity, cost, and margin assessment limited to the posterolateral margin [7,16,17,18] (Table 1).

Clinical perspective: NeuroSAFE remains the most clinically established intraoperative margin-assessment strategy during RARP and currently serves as the benchmark against which emerging techniques should be judged. Its main limitation is not proof of concept, but the infrastructure, cost, and workflow required for routine implementation.

### 3.2. Fluorescence Confocal Microscopy

Confocal laser scanning microscopy (CLSM) produces optical sections by scanning a focused laser and rejecting out-of-focus light with a detector pinhole, thereby sampling a small tissue volume [21]. In reflectance CLSM, contrast derives from backscattered photons generated by microscopic refractive-index differences [21]. In fluorescence CLSM, commonly termed fluorescence confocal microscopy (FCM), fresh tissue is stained with a fluorescent dye, which highlights nuclei upon laser excitation and improves their visualization relative to the surrounding stroma [22]. FCM is an optical technique for examining freshly excised tissue using combined fluorescence and reflectance imaging, generating digital images that resemble histological cross-sections, usually within minutes. In prostate specimens, FCM can depict non-neoplastic connective, muscular, fatty, and neural tissue, while preserving the sample for subsequent conventional histopathology and immunohistochemistry. Its digital format also enables remote pathological interpretation and may facilitate future artificial intelligence (AI)-based analysis [23,24].

Rocco et al. described a Mohs-like technique for intraoperative FCM assessment during RARP. After intrafascial prostate removal, the specimen is oriented with the apex toward the surgeon, with the base and seminal vesicles on the opposite side. Three shaved margins are then obtained (apex, right posterolateral, left posterolateral). Each sample is dipped in acridine orange for 30 s, rinsed in saline, placed between two glass slides, and positioned on the VivaScope scanner [23]. This technique still requires sectioning of the specimen. The main advantage is the avoidance of freezing and the immediate digital readout (Figure 2).

LaserSAFE is closely related to FCM but uses a distinct Histolog scanner for en face assessment of the intact prostate specimen. In the feasibility study by Almeida-Magana et al., the excised gland was stained with Histolog Dip, and three surfaces—the posterior, right posterolateral, and left posterolateral surfaces—were scanned directly in the operating theatre before NeuroSAFE processing [25]. This distinguishes LaserSAFE from the Mohs-like shaving approach described by Rocco et al. [23]. Most, but not all, studies support a time advantage over intraoperative frozen section (IFS), while diagnostic performance remains promising but heterogeneous and depends on factors such as reader experience, diagnostic thresholds, scanning completeness, and local workflow (Table 2). Diagnostic performance also appears to depend substantially on the minimum margin length considered clinically relevant. In the LaserSAFE study, sensitivity increased from 71% for all positive margins to 91% when margins ≥ 0.5 mm were considered [26]. In the larger series by Mayor et al., sensitivity increased from 48% for all positive margins to 79% for margins ≥ 3 mm, with lower performance for small and apical margins [27]. This indicates that diagnostic accuracy estimates are highly dependent on the definition and length threshold used for a positive margin and should therefore be interpreted cautiously when comparing studies. Cross-study comparisons are further complicated by differences in pathological processing and reference standards. FCM findings have been compared with frozen-section protocols, paraffin-embedded histopathology, and whole-mount histology, and these differences in specimen processing and sampling may influence whether small or focal margin involvement is detected and therefore affect reported sensitivity and specificity.

Despite its potential for rapid digital margin assessment, the clinical reliability of FCM remains limited by heterogeneous diagnostic performance across studies. In particular, variable sensitivity, especially for short and apical margins, raises concern for false-negative findings and limits its reliability as a stand-alone intraoperative decision-making tool. IFS generally retains higher diagnostic performance when directly compared, while workflow turnaround time remains center-dependent [24,25,26,28,29]. This variability is illustrated in the study by Skok et al., in which the longer turnaround likely reflected the full workflow, including specimen transfer, pathology handling, prospective interpretation, and confirmatory frozen section in suspicious cases, instead of slower image acquisition itself [29].

From an implementation perspective, wider adoption of FCM will require reproducible specimen handling and scanning workflows together with consistent criteria for image interpretation. The need for dedicated equipment and trained readers, combined with center-dependent workflow and variable sensitivity for small margins, currently limits routine implementation.

### 3.3. PSMA-Targeted Molecular Margin Assessment

PSMA-targeted techniques aim at cancer localization and margin assessment using nuclear medicine modalities. Currently, positron-emitting radionuclides such as ^68^Ga and ^18^F coupled with PSMA tracers are used to detect cancer cells at the tumor margin. However, a fundamental limitation of these approaches is that they detect PSMA-avid disease (tumor biology) rather than the true cellular-level histological margin status. Consequently, these techniques often identify tumor cells in close proximity to the margin, which must be clearly distinguished from a histologically confirmed positive surgical margin (where the tumor is actively transected). In order to visualize radiation from cancer-marker hotspots, the most frequently utilized methods are Cerenkov Luminescence Imaging (CLI) and PSMA PET/CT.

CLI uses Cerenkov radiation, which is produced by charged particles (beta-particles) from isotopes, e.g., ^18^F and ^68^Ga, that exceed the phase velocity of light in a dielectric medium such as tissue. When the polarized medium along the path of the particle returns to its ground state, it emits electromagnetic radiation. Because Cerenkov radiation has very low intensity, it can only be detected by high-sensitivity optical equipment in a dark environment [30].

The typical ^68^Ga-PSMA CLI workflow involves ex vivo imaging of the prostate specimen after removal. Depending on the protocol, approximately 100 MBq of ^68^Ga-PSMA is injected immediately after docking of the da Vinci robot. After prostate excision, the specimen is washed with sodium chloride to reduce potential contamination from blood and urine. Dedicated CLI systems, such as LightPath^®^ (Lightpoint Medical Ltd., Chesham, UK), are then used to image six surfaces of the prostate: left, right, anterior, posterior, basal, and apical. Images are usually acquired with 150–300 s of exposure and 8 × 8 pixel binning. If an increased signal is detected on the specimen surface, an additional image can be obtained using a 550 nm short-pass filter, which has been reported to reduce false-positive signals [30,31].

Clinical results with ^68^Ga-PSMA CLI suggest feasibility but also important limitations. In the study by Olde Heuvel et al., overall agreement between ^68^Ga-PSMA CLI and histopathology was relatively low at 31%, although it improved to 59% when close surgical margins, defined as ≤1 mm, were included. CLI correctly identified PSMs at the apex and midgland, but a high interfering signal at the prostate base contributed to false-negative findings. False-positive signals also remain a major limitation. Extensive rinsing with sodium chloride did not eliminate these signals, and diathermy-related artifacts have been suggested as a possible cause. In addition, CLI provides limited depth information, which complicates the precise localization of the signal source [30].

^18^F-PSMA was also evaluated for intraoperative margin assessment with a similar workflow to that for ^68^Ga, using standard CLI and flexible autoradiography-enhanced CLI (FAR CLI) on RP specimens; standard CLI showed no increased signal over the index lesion, whereas FAR CLI improved tumor signal detection and visualized the index lesion in six of seven patients, but neither CLI nor FAR CLI detected the two histopathologically positive resection margins. ^18^F has a theoretically 26-fold lower Cerenkov light yield than ^68^Ga due to its lower β+ energy, and in real biological tissue, the signal is further weakened by absorption, scattering, and imperfect anatomic correlation because the scintillator in FAR overlies the specimen, thereby limiting accurate margin localization [32,33].

PSMA-targeted imaging is not limited to CLI, and dual-modality probes may offer an alternative approach. Chen et al. evaluated a dual-modality PSMA-targeted probe (^68^Ga-P3) that combines preoperative PET/CT with intraoperative fluorescence guidance during RARP. Sixteen patients underwent PET/CT 30–120 min after probe administration and surgery 24 ± 6 h later using sensitive Firefly fluorescence imaging, with suspicious fluorescent regions correlated with histopathology. Intraoperative fluorescence achieved an NPV of 100%, a PPV of 43.8% and an overall accuracy of 90.9%. However, this figure can be misleading if strictly interpreted as the detection of histologically confirmed PSMs. In reality, this correlation primarily reflected the detection of PSMA-expressing tumor tissue adjacent to the margin. Importantly, most false-positive fluorescent sites (88.9%) corresponded to tumors adjacent to the surgical margin, underscoring the clinically relevant distinction between detecting tumor proximity and confirming actual margin positivity [34].

Ex vivo PSMA PET/CT provides another strategy by enabling 3D assessment of the resected prostate specimen. Unlike CLI, which relies on low-intensity optical signals from superficial tissue layers, intraoperative ex vivo PSMA PET/CT enables 3D assessment with direct CT anatomic correlation. Using ^18^F-PSMA-1007 and dedicated specimen PET/CT imaging with semiautomatic segmentation, this method achieved 83% sensitivity, 100% specificity, 100% PPV, 92% NPV, and 94% accuracy for PSM detection, although it remained limited to macroscopic PSMA-positive lesions [35].

To date, none of the currently available clinical studies of PSMA-based intraoperative imaging have evaluated or demonstrated an effect on nerve-sparing decisions or postoperative functional outcomes; the available evidence remains focused on tumor localization and surgical margin detection [30,31,32,34,35].

### 3.4. Structured Illumination Microscopy

Structured illumination microscopy (SIM), particularly video-rate SIM (VR-SIM), enables high-resolution circumferential imaging of the intact prostate surface without Mohs-like sectioning. It offers panoramic visualization of the anterior, posterior, and lateral surfaces of the prostate [36]. In early feasibility work, VR-SIM identified most positive circumferential margins on final pathology and detected additional margin involvement not recognized by standard histopathological examination [36]. The highest available evidence is limited to ex vivo clinical feasibility studies; among these, Luethy et al. introduced an Automated Prostate Positioning System (APPS) that enabled automated continuous circumferential imaging from apex to base and improved surface coverage and workflow [37]. However, diagnostic performance has not yet been prospectively validated in a clinical intraoperative setting. Thus, the principal barrier to clinical translation remains the lack of prospective evidence demonstrating reproducible diagnostic accuracy and integration into real-time intraoperative decision-making [36,37].

### 3.5. Raman Histology

Stimulated Raman histology (SRH) is a label-free optical imaging technique that generates high-resolution images of fresh, unprocessed tissue. It uses the intrinsic vibrational signatures of tissue components such as lipids, proteins, and nucleic acids and can produce images that resemble H&E specimens without fixation, sectioning, or staining [38,39,40,41]. Clinical translation of SRH has been enabled by portable fiber-laser-based stimulated Raman scattering systems where SRH mosaics are acquired in about 2.5 min and can be transmitted directly from the operating room to a networked workstation [42]. SRH has shown promising results in distinguishing PCa from benign tissue. In a study by Mannas et al., unprocessed prostate biopsies were prospectively imaged, and a convolutional neural network was trained on 303 biopsies from 100 participants. On an independent test set of 113 biopsies, AI-based interpretation of SRH achieved 96.5% accuracy, 96.3% sensitivity, and 96.6% specificity for PCa detection relative to conventional pathology [43].

In the intraoperative setting, the prospective ROBOSPEC pilot study evaluated AI-enhanced SRH for margin assessment during RARP in 18 patients with intermediate- or high-risk PCa. Bilateral dorsolateral margin samples were obtained from areas adjacent to the NVBs and compared with conventional IFS hematoxylin and eosin analysis. From 33 evaluable samples, 141 SRH images were generated, with a mean image-generation time of 8.2 min per sample. An AI algorithm identified PSMs in 4/18 patients (22%), whereas cryo-H&E identified them in 3/18 (17%); patient-based analysis showed sensitivity and NPV of 1.00, specificity of 0.93, and PPV of 0.75, while sample-based specificity was 0.97. Given the very high NPV and the anatomic relevance of dorsolateral margins for NS surgery, SRH may be particularly useful for intraoperative decision-making during RARP [44]. However, broader intraoperative translation remains limited by the small preliminary clinical cohort and by the need for independent validation of the AI-based interpretation. In particular, it remains uncertain whether the reported performance can be reproduced across different centers, imaging systems, or patient populations. Further studies should therefore assess external validity and concordance with expert histopathology before AI-assisted SRH can be incorporated into routine intraoperative decision-making [44].

### 3.6. OCT

Optical coherence tomography (OCT) provides cross-sectional near-infrared imaging of tissue microarchitecture and can visualize structures such as nerves, vessels, adipose tissue, and stromal planes. The highest available evidence for OCT surgical margin assessment is a single-center clinical feasibility study of 100 radical prostatectomy specimens, in which OCT findings were compared with final histopathology. OCT demonstrated a sensitivity of 70%, specificity of 84%, positive predictive value of 33%, and negative predictive value of 96% for PSM detection [45]. Despite the relatively high negative predictive value, the principal barrier to clinical translation remains limited validation for reliable real-time intraoperative margin assessment [45,46,47]. Consequently, OCT remains investigational and has no established role in routine margin assessment during RARP.

### 3.7. Reflectance Spectroscopy

Reflectance-based optical techniques, including diffuse reflectance spectroscopy, can distinguish malignant from benign prostate tissue by analyzing tissue-specific absorption and scattering signatures [48,49,50]. Ex vivo studies have shown encouraging diagnostic accuracy, especially for clinically significant positive margins [51]. However, translation into intraoperative use remains uncertain because most available models are derived from ex vivo spectra and may not be directly applicable to living tissue [52].

### 3.8. MPM

Multiphoton microscopy enables high-resolution optical sectioning of fresh tissue and can visualize both glandular and stromal structures, including periprostatic anatomy [53,54,55]. Experimental studies have demonstrated feasibility in ex vivo human prostate specimens, and endoscopic adaptation appears technically possible [56]. However, no validated diagnostic performance metrics, including sensitivity or specificity for positive surgical margin detection, have yet been reported. Accordingly, the principal barrier to clinical translation is the absence of clinical validation for real-time intraoperative margin assessment.

From a clinical perspective, diagnostic accuracy metrics should be interpreted according to their consequences for intraoperative decision-making. High sensitivity and negative predictive value are particularly important when a negative result is used to support preservation of the neurovascular bundle, because a false-negative result may provide inappropriate reassurance in the presence of a true positive margin. Conversely, limited specificity or positive predictive value may lead to unnecessary secondary neurovascular resection, potentially compromising postoperative functional recovery. Therefore, clinical utility depends not only on diagnostic accuracy but also on whether test-guided changes in surgical management reduce clinically meaningful positive margins while preserving erectile and urinary function. With the exception of NeuroSAFE, such downstream patient-level benefits have not yet been established for most emerging margin-assessment technologies.

Taken together, these modalities remain technologically attractive but clinically immature, and for most of them the available evidence is still dominated by ex vivo feasibility studies or early clinical validation rather than workflow-integrated studies demonstrating reproducible intraoperative benefit. In this context, reported diagnostic performance should be interpreted according to its consequences for surgical decision-making. Sensitivity reflects the ability of a technique to detect a truly positive margin, whereas specificity reflects its ability to correctly identify a negative margin. Accordingly, limited sensitivity may result in false-negative findings and inappropriate preservation of the neurovascular bundle despite true margin involvement, while limited specificity may increase false-positive findings and prompt unnecessary secondary neurovascular resection. Positive predictive value indicates how likely a positive intraoperative result is to represent a true positive margin, whereas negative predictive value indicates how reliably a negative result excludes margin involvement. Thus, the clinical value of an intraoperative margin-assessment technique depends not only on diagnostic accuracy but also on whether its use translates into improved margin control without compromising erectile or urinary outcomes.

At present, such patient-level benefit has been demonstrated most convincingly for NeuroSAFE, whereas evidence for emerging optical and molecular techniques remains largely diagnostic or feasibility-based.

## 4. Intraoperative Surgeon-Guidance Tools

### 4.1. Intraoperative Ultrasonography

Intraoperative ultrasonography during RARP should currently be regarded primarily as a navigation tool rather than as a surgical margin-assessment technique. Early feasibility studies demonstrated that a robotically manipulated biplanar transrectal ultrasound probe could provide real-time visualization of key anatomic structures like the bladder neck and NVBs, and it also allowed identification of color Doppler arterial flow and biopsy-confirmed hypoechoic lesions, allowing the surgeon to selectively widen dissection [57]. In a series of ten patients with intraoperative transrectal ultrasonography (TRUS), NS was performed in nine patients, and PSMs were detected in one patient. Detection of biopsy-proven hypoechoic lesions was reported to alert the surgeon to perform wider margin excision around these lesions. Moreover, 43% (3 out of 7) of men with preoperative erectile function preserved their erections, and 8 out of 10 reported urinary continence (1 pad per day) after a median 9-week follow-up [57]. Further trials included a surgeon-manipulated drop-in ultrasound microtransducer: In 10 patients, localization of biopsy-proven hypoechoic cancer was feasible in 4/4 visible lesions; the ultrasound imaging guided complete versus partial NVB excision, and negative margins were achieved in all patients despite 50% pT3 disease; still, the method remained limited by the 9 mm scan depth, difficulty in visualizing the full extent of cancer, and the very small sample size [58]. Overall, the highest level of evidence in the available ultrasound literature remains small prospective or case-series experience; thus, intraoperative ultrasonography appears promising for intraoperative topographic guidance, but it has not been incorporated into standard workflow for margin assessment in RARP and is used mainly to localize tumor or tumor-adjacent anatomy.

### 4.2. Three-Dimensional Models and Augmented Reality Navigation

Among intraoperative guidance methods, the literature on 3D models and AR provides the largest body of evidence for intraoperative guidance to support NS and reduce PSMs. These models are usually generated from mpMRI images, sometimes enriched with biopsy-core information or PSMA PET/CT data, and displayed either cognitively on a separate screen or as AR overlays on the robotic console. They help visualize the index lesion, capsular contact, EPE, sphincter, and NVBs. Recent studies focus on the use of 3D models during the extirpative phase of surgery to widen the dissection areas around sites of capsular proximity to the tumor or EPE. Most published studies report manually oriented 3D models, with newer studies reporting AI-assisted, overlaid 3D models.

Early feasibility studies established the technical basis for 3D/AR navigation during RARP. Ukimura et al. used 3D TRUS/MRI-guided biopsy data to reconstruct a navigation model displayed on the da Vinci console and manually aligned with the operative field. The model integrated the prostate, biopsy-proven index lesion, NVBs, urethra, and biopsy trajectories. Index-lesion location in the software-created model correlated with final histology, and negative margins were achieved in most cases, except in one case with extensive EPE. However, gray-scale TRUS underestimated tumor volume, whereas mpMRI-based 3D models were more accurate when compared with final histopathology [59].

Subsequent studies evaluated mpMRI-based AR models for lesion localization and intraoperative decision-making. High-resolution mpMRI-derived 3D models enabled lesion marking and selective biopsy of suspected EPE, with positive AR-guided biopsies in most cT3 cases and no PSMs in pT2 disease; however, manual orientation and rigid registration remained important limitations [60]. AR-3D models generated by fusing mpMRI with ^68^Ga-PSMA PET/CT appeared particularly useful in pT3 disease, especially for achieving negative apical margins and informing side-specific NS decisions, although extensive pT3 disease was still associated with PSMs [61].

More advanced AR approaches attempted to account for prostate deformation. Elastic AR, manually adjusted according to real-time prostate deformation, improved dynamic lesion tracking during dissection and more accurately identified capsular involvement than 2D cognitive imaging. Although the AR group had fewer PSMs, the difference was not statistically significant, and cancer in suspicious areas was confirmed microscopically in 95.4% of cases [62].

Clinical studies further assessed whether AR-3D guidance could improve NS decisions and margin outcomes. Schiavina et al. showed that mpMRI-based real-time AR-3D changed planned NS strategy in a substantial proportion of patients and prostate sides, with high appropriateness (94.4%) and good lesion-localization accuracy (92%) [63]. AR-3D has also been used to target IFS at the index lesion; in a propensity-matched analysis, overall PSM rates were similar, but index-lesion PSMs were significantly reduced (20% vs. 5%) [64].

The benefit of 3D models may also extend to centers already using intraoperative margin-assessment workflows. In a prospective series, integration of mpMRI-derived 3D models into the robotic console in a center already using NeuroSAFE significantly reduced PSMs on frozen section from 22.5% to 11.3% and on final pathology from 13.1% to 6.6% (*p* ≤ 0.03). This suggests that 3D models may improve margin control even in an already image-informed workflow [65]. Similarly, a larger prospective comparison of 160 patients managed with 3D models and 640 controls showed lower overall PSM rates in the 3D group (25.0% vs. 35.1%, *p* = 0.01), together with a shift toward a more NS approach. It was also indicated that 3D model availability was independently associated with a lower risk of PSMs, with the strongest association observed in the EPE and pT3 subgroups [66].

Recent studies have focused on AI-assisted and automatically overlaid AR-3D models, which reduce the need for external manual assistance during surgery. In a study by Checcucci et al., selected patients had an mpMRI-visible index lesion with capsular contact or EPE, and the 3D model was overlaid at the end of the extirpative phase to guide excisional biopsy of the NVBs. The biopsy detected a tumor on the spared NVB in 87.5% of pT3 patients, with PSM rates of 0% in pT2 and 7.1% in pT3 disease. Functional outcomes were also promising, with 12-month recovery rates of 94.1% for urinary continence and 70% for erectile function [67].

The highest level of evidence comes from the randomized RIDERS trial, which compared cognitive MRI guidance with 3D-AI-AR guidance. In this trial, 133 men with bulging or extracapsular extension on MRI were randomized in a 2:1 ratio. A total of 136 patients were randomized, of whom 133 received the allocated intervention; therefore, the reported analysis was not based on a strict intention-to-treat population.

After prostate excision, additional NVB excisions were performed in both the cognitive and 3D guidance groups. Compared with cognitive MRI guidance, 3D-AI-AR guidance was associated with a lower overall PSM rate (22% vs. 39%, *p* = 0.047), higher positivity of selective biopsies at preserved NVBs (52% vs. 13%), less postoperative radiotherapy (18% vs. 35%), and better 12-month continence recovery (91% vs. 71%). No significant differences were observed in potency or biochemical recurrence [68]. As no adjustment for multiplicity was reported for the secondary endpoints, these secondary findings should be interpreted as exploratory.

Moreover, the current evidence for 3D/AR-guided RARP derives predominantly from a limited number of highly experienced, high-volume centers. This also applies to the RIDERS trial, which, despite its multicenter randomised design, was conducted at only two Italian centers with substantial experience in 3D/AR-guided robotic surgery. Consequently, the generalisability of these findings to lower-volume centers and routine clinical practice remains uncertain and requires external multicenter validation.

Clinical perspective: 3D/AR navigation currently has the strongest supportive evidence among surgeon-guidance tools, with the RIDERS randomized trial demonstrating significantly lower PSM rates and improved continence compared with cognitive MRI guidance. Automatic AI-based overlay reduces the need for external manual assistance, but its broader clinical use is still limited by uncertainty about how well current algorithms will perform outside the centers in which they were developed. Differences in image acquisition and model construction can affect the final overlay and may reduce reproducibility when the technology is transferred to other settings. This makes independent multicenter validation particularly important before these systems can be considered suitable for routine use. In parallel, regulatory approval should be regarded as a separate step from clinical feasibility, because favorable results in early studies do not by themselves establish that an AI-assisted system is authorized for routine intraoperative use.

### 4.3. Intraoperative Fluorescence

Indocyanine green (ICG), used with near-infrared fluorescence, is primarily used to identify the landmark artery and the vascularized planes of the NVBs. The available literature focuses mainly on NVB identification, allergic reactions, and erectile function. ICG has been reported to achieve NVB identification rates of up to 100%, with no allergic reactions and preservation of preoperative baseline erectile function. However, the available evidence is limited to single-arm retrospective studies, with no data on margin status [69,70].

### 4.4. Image-Enhancing Techniques

The studies on image-enhancement techniques in RARP are preclinical and provide only retrospective validation. They point to the promising concept that the surgical video itself can be processed to magnify subtle pulsatile motion and reveal arteries or NVBs. McLeod et al. proposed motion magnification as a workflow-friendly method with low complexity that enhances pulse-synchronous intensity changes in endoscopic video; in RARP, the approach made very subtle pulsation in the region of the NVBs more conspicuous, with the objective of aiding vessel identification, but they reported no oncological, functional, or diagnostic endpoints [71]. Fan et al. later advanced this concept using hybrid temporal filtering and deeply learned spatial decomposition; in RARP videos, their method outperformed comparator magnification strategies on quantitative image-quality metrics. Six surgeons subjectively agreed that it could assist in the identification of vessels and NVBs. Nonetheless, the magnification factor remained empirically chosen, and the model was not trained on surgical videos [72]. Pulsation-enhancement techniques remain a promising visualization aid for NS dissection and, at present, have not been incorporated into standard robotic prostatectomy workflows.

## 5. Conclusions

Intraoperative margin assessment and surgeon-guidance methods may help balance oncological control with nerve preservation during RARP, but the strength of evidence differs substantially between techniques. The absence of a formal risk-of-bias assessment is an important limitation of this narrative review and should be considered when interpreting comparisons across the available evidence.

NeuroSAFE remains the most established method, with the strongest clinical evidence for reducing PSMs and supporting NS. FCM/LaserSAFE has progressed beyond initial feasibility studies, but its heterogeneous diagnostic performance and limited evidence of patient-level benefit currently preclude routine implementation. PSMA-targeted molecular techniques should also be regarded as investigational margin-assessment approaches. Ultrasound, fluorescence guidance, and image-enhancement systems remain guidance tools, while 3D/AR navigation has the strongest supportive evidence within this group.

However, improved margin control should not be interpreted as evidence of improved long-term cancer control. For NeuroSAFE, longer-term observational data provide reassurance that the technique does not appear to compromise oncological outcomes, but evidence that it improves biochemical recurrence-free or survival outcomes remains insufficient. For 3D/AR navigation and the other emerging technologies reviewed here, the evidence is even less mature and is largely limited to margin status and other short-term endpoints. Long-term prospective data are therefore required before an oncological benefit beyond improved margin control can be established.

For most emerging technologies, external validation remains limited, and it is therefore uncertain whether the reported performance can be reproduced in routine practice outside the centers in which these methods were developed. This concern is particularly relevant for FCM, where diagnostic sensitivity varies considerably between published series and appears to depend on margin definition, reader experience, and local workflow. AI-enhanced SRH has so far been evaluated intraoperatively only in a small preliminary cohort, while several other optical approaches remain supported mainly by single-center or ex vivo studies. Although 3D/AR navigation has a stronger clinical evidence base, much of the literature still originates from a limited number of highly experienced centers, and even the randomized RIDERS trial was conducted in only two specialist institutions. These findings demonstrate feasibility in selected settings but do not establish that comparable performance can be achieved across different institutions and routine surgical settings. Independent multicenter validation is therefore needed before broader generalizability and widespread clinical implementation can be assumed.

Generalizability to lower-volume centers remains an important concern. Many of the favorable results reviewed here were obtained in experienced institutions with established robotic programs and access to dedicated pathology and imaging technologies. The same outcomes may therefore not be readily reproducible in centers where case volumes are lower and such resources are less accessible. This is particularly relevant for NeuroSAFE and advanced 3D/AR workflows, while the center-dependent performance and workflow reported for FCM raise similar concerns. Evaluation in less specialized settings will be important before these approaches can be considered broadly applicable to routine RARP.

In terms of translational readiness, NeuroSAFE has the most mature clinical evidence and is already applicable in centers with the necessary pathology infrastructure. 3D/AR navigation has the strongest evidence among surgeon-guidance tools, although its broader applicability outside experienced centers remains uncertain. FCM/LaserSAFE requires further multicenter validation and standardization of diagnostic thresholds and reader training before routine implementation. Specifically, achieving this standardization will require the development of consensus-based digital pathology atlases, the establishment of uniform cut-off values for margin positivity (e.g., defining the exact depth or length of tumor contact that mandates further resection), and the implementation of structured, multi-institutional certification programs for urologists and pathologists interpreting these novel optical images. SRH with AI analysis remains at an early stage of clinical evaluation. The other emerging optical, molecular, fluorescence, ultrasound, and image-enhancement approaches should continue to be regarded as investigational until more robust prospective clinical evidence becomes available (Table 3).

## Figures and Tables

**Table 1:** Practical and methodological limitations of NeuroSAFE for intraoperative margin assessment during RARP.

NeuroSAFE Limitations	Reference
Requires a cryostat pathology setup and dedicated staff during surgery, usually 2 cryostats, 3 technicians, one pathologist (occasionally two to reach consensus).	[16]
Prolonged intraoperative workflow—data are inconsistent. Some studies report prolonged times. Dinneen et al. report 196 min (NeuroSAFE) vs. 134 min (control), and some report no difference.	[17,19]
Additional cost: €626 per procedure (2023 price levels; Dutch healthcare perspective; base-case assumption of 150 procedures/year)	[18]
Limited intraoperative margin assessment—only posterolateral margins	[7]
Specimen artefacts due to dissection and freezing	[20]
False positive surgical margins due to mistaking artificial and true margins of the posterolateral prostatic tissue	[16]

**Table 2:** Studies evaluating fluorescence confocal microscopy for intraoperative surgical margin assessment during robot-assisted radical prostatectomy, including reference standards, diagnostic performance, inter-reader agreement, and workflow time. NR—not reported, IFS—intraoperative frozen section, FCM—fluorescence confocal microscopy, RARP—robot-assisted radical prostatectomy, NPV—negative predictive value, PPV—positive predictive value, PSM—positive surgical margin, IQR—interquartile range.

Author, Year	Device Used for Prostate Scanning	Reference Standard	Study Sample	Diagnostic Performance of FCM for Positive Surgical Margins	Diagnostic Performance of Frozen Section Method for Positive Surgical Margins	Interreader Agreement (Kappa)	Time for FCM vs. Frozen Section Method
Baas et al., 2023 [28]	Histolog Scanner	NeuroSAFE and definitive histopathology	50 RARP patients evaluated; 1 excluded; final analysis 49 patients and 96 posterolateral sides (47 left, 49 right)	Sensitivity 86%, specificity 96%, PPV (positive predictive value) 80%, NPV (negative predictive value) 98%	NeuroSAFE: sensitivity 93%, specificity 99%, PPV 93%, NPV 99%	Cohen’s kappa = 0.80	Median 8 min (FCM) vs. 50 min (NeuroSAFE)
Musi et al., 2024 [24]	VivaScope scanner	Final pathology, comparison with IFS	45 patients; 54 margins; 2 FCM readers	Best FCM result (experienced pathologist, Definition C): sensitivity 70.5%, specificity 91.8%, PPV 80.0%, NPV 87.1%	IFS: sensitivity 88.2%; specificity 100%, PPV 100%, NPV 94.8%	Inter-observer kappa for FCM = 0.74–0.90, agreement between FCM and IFS = 0.62–0.86	NR
Almeida-Magana et al., 2024 [25]	Histolog Scanner	Paraffin-embedded analysis	31 prostate specimens; lateral side analysis, 11 paraffin-positive margins, 4 blinded pathologists	Sensitivity 73–91%; specificity 94–100%, PPV 77–100%, NPV 94–98% (range across 4 readers)	NR	Fleiss’ kappa for PSM detection = 0.78 (95% CI 0.64–0.92), kappa for NVB resection recommendation = 0.748	Acquisition about 50 s per side, no frozen section comparator reported
Almeida-Magana et al., 2025 [26]	Histolog Scanner	NeuroSAFE and final histopathology	20 patients, right and left sides assessed (20 per side)	All margins: sensitivity 71%, specificity 100%, PPV 100%, NPV 87%. Margins ≥ 0.5 mm: sensitivity 91%; specificity 100%, PPV 100%, NPV 97%	NeuroSAFE diagnostic metrics not separately reported; study states all NeuroSAFE-identified PSMs were concordant with final paraffin histopathology	Cohen’s kappa 0.76 for all margins, 0.94 for margins ≥ 0.5 mm, secondary resection recommendation kappa 0.79	Median 7 min (LaserSAFE) vs. 63 min (NeuroSAFE)
Mayor et al., 2026 [27]	Histolog Scanner	Final histopathology	156 RP/RARP patients, whole prostate surface scanned en face; 869 evaluable surfaces	For all PSMs: sensitivity 48%, specificity 94%, PPV 79%, NPV 80%. For PSMs ≥ 3 mm: sensitivity 79%, specificity 94%, PPV 71%, NPV 96%. Performance was lower for small and apical margins.	NR	NR	NR
Skok et al., 2026 [29]	Histolog Scanner	Whole-mount histology, comparison with frozen section	68 enrolled, 2 excluded; 66 evaluable cases	Definite calls only: sensitivity 60.0%, specificity 98.1%, PPV 75.0%, NPV 96.4%. Suspicious counted as positive: sensitivity 75.0%, specificity 91.4%, PPV 54.5%; NPV 96.4%	NR	NR	Median diagnosis time 30 min (IQR 24–41) for Histolog workflow vs. 25 min (IQR 20–30) for retrospective local frozen section cohort

**Table 3:** Current evidence, limitations, and clinical readiness of intraoperative margin-assessment and surgeon-guidance techniques during RARP. To improve the reproducibility of the translational-readiness assessment, each technology was additionally mapped to the IDEAL framework according to the highest level and breadth of available evidence, ranging from Stage 0 (preclinical), Stage 1 (Idea/first-in-human), Stage 2a (Development), Stage 2b (Exploration), Stage 3 (Assessment, typically an RCT), to Stage 4 (long-term surveillance) [73]. AI—artificial intelligence; AR—augmented reality; CLI—Cerenkov luminescence imaging; CT—computed tomography; EPE—extraprostatic extension; FCM—fluorescence confocal microscopy; ICG—indocyanine green; NeuroSAFE—neurovascular structure-adjacent frozen-section examination; PET—positron emission tomography; PET/CT—positron emission tomography/computed tomography; PSMA—prostate-specific membrane antigen.

Technique	Main Role	Best Current Evidence	Main Limitation	Readiness for Clinical Use
Direct intraoperative margin assessment				
NeuroSAFE	Frozen-section intraoperative margin assessment	Randomized controlled trial	Dedicated pathology infrastructure, adds workflow complexity	IDEAL 3 (Assessment): clinically established in experienced centers with appropriate infrastructure
FCM/LaserSAFE	Confocal microscopy-based intraoperative margin assessment	Prospective, blinded, paired-cohort trial	Heterogeneous diagnostic sensitivity, particularly for short and apical margins; performance appears better for longer margins; costly equipment and requirements for reader training and workflow standardization	IDEAL 2b (Exploration): clinically promising; further multicenter validation and standardization required
PSMA-targeted molecular margin assessment	Tumor localization and margin assessment using CLI, fluorescence, or PET/CT	Early feasibility studies of PSMA CLI, dual-modality probes, and ex vivo PSMA PET/CT	High false-positive rates, limited depth information, incomplete margin validation	IDEAL 2a (Development): investigational
Structured Illumination Microscopy	High-resolution (circumferential) imaging of the prostate surface	Ex vivo proof-of-concept studies	No validated intraoperative workflow, limited evidence, incomplete assessment of apex and base	IDEAL 0 (Preclinical): investigational
Stimulated Raman Histology	Label-free optical histology of fresh tissue	Prospective single-arm pilot study	Very early clinical evidence, small sample sizes, requires AI, pathology validation and workflow integration	IDEAL 2a (Development): investigational
Optical coherence tomography	Cross-sectional optical imaging of tissue microarchitecture	Early clinical feasibility studies	Moderate diagnostic performance, limited validation for real-time margin assessment	IDEAL 2a (Development): investigational
Reflectance spectroscopy	Optical tissue characterization based on absorption and scattering signatures	Ex vivo feasibility studies	Mostly ex vivo evidence; uncertain translation to living intraoperative tissue	IDEAL 0 (Preclinical): investigational
Multiphoton microscopy	Optical imaging of fresh prostate and periprostatic tissue structures	Ex vivo feasibility studies	No clinical evidence for real-time intraoperative margin assessment	IDEAL 0 (Preclinical): investigational
Surgeon-guidance tools				
Intraoperative ultrasonography	Real-time anatomic localization of confirmed lesions and guidance during dissection	Small prospective series and feasibility studies	Limited sample size and tumor extent visualization	IDEAL 2a (Development): investigational
3D/AR navigation	Augmented reality surgical navigation to support side-specific nerve-sparing and wider excision near suspected EPE or tumor contact	Randomized trial and prospective propensity-matched trials	High-quality imaging, appropriate software, AR infrastructure, registration accuracy, and surgeon training	IDEAL 3 (Assessment): clinically promising in specialized centers; external validation required
ICG near-infrared fluorescence	Visualization of landmark artery and neurovascular bundle vascular planes	Single-arm retrospective studies	Mainly supports anatomical identification, no margin assessment evidence	IDEAL 2a (Development): limited guidance tool
Image-enhancement techniques	Digital enhancement of subtle pulsation to identify vessels and neurovascular bundles	Preclinical and retrospective validation studies on recorded video material	No oncological, functional, or diagnostic endpoint validation, not integrated into standard workflow	IDEAL 0 (Preclinical): investigational

## Data Availability

No new data were created or analyzed in this study. Data sharing is not applicable to this article.
